# Complex Odontome associated with Maxillary Impacted Permanent Central Incisor: A Case Report

**DOI:** 10.5005/jp-journals-10005-1189

**Published:** 2013-04-26

**Authors:** Raghavendra M Shetty, Sangamesh Halawar, Hanumanth Reddy, Sujata Rath, Sunaina Shetty, Anushka Deoghare

**Affiliations:** Professor, Department of Pedodontics and Preventive Dentistry Chhattisgarh Dental College and Research Institute, Rajnandgaon Chhattisgarh, India; Associate Professor, Department of Oral Pathology, KSR Institute of Dental Science and Research, Tiruchengode, Tamil Nadu, India; Professor, Department of Orthodontics, Chhattisgarh Dental College and Research Institute, Rajnandgaon, Chhattisgarh, India; Professor, Department of Pedodontics and Preventive Dentistry Chhattisgarh Dental College and Research Institute, Rajnandgaon Chhattisgarh, India; Assistant Professor, Department of Periodontics, Chhattisgarh Dental College and Research Institute, Rajnandgaon, Chhattisgarh, India; Postgraduate Student, Department of Pedodontics and Preventive Dentistry, Chhattisgarh Dental College and Research Institute Rajnandgaon, Chhattisgarh, India

**Keywords:** Complex odontome, Impacted incisor, Ligature hook

## Abstract

Odontomas, the most often seen ones among odontogenic tumors, are usually asymptomatic and discovered in routine radiographic examinations. Frequently it may interfere with the eruption of teeth. The purpose of this article is to present and discuss the case of a 12-year old child with a complex odontome, obstructing the eruption of left maxillary permanent central incisor. Radio opaque calcified masses were revealed in the radiograph and the masses were surgically removed to facilitate the eruption of the tooth.

**How to cite this article:** Shetty RM, Halawar S, Reddy H, Rath S, Shetty S, Deoghare A. Complex Odontome associated with Maxillary Impacted Permanent Central Incisor: A Case Report. Int J Clin Pediatr Dent 2013;6(1):58-61.

## INTRODUCTION

An odontoma is a mixed tissue benign tumor of odontogenic origin, which exhibits complete dental tissue differentiation. Complex odontome constitute about 5 to 30% of all odontogenic tumors of the jaws.^1^ They are usually detected in school-age children, and the mean age at the time of diagnosis is 14 years.^2,3^ The majority of odontomes are asymptomatic and seldom cause swelling, pain, suppuration, bony expansion and displacement of teeth.^2,4^ These lesions are commonly small, seldom larger than a tooth. Odontomes are generally discovered through routine radiographic examination in dental treatment. An odontome is essentially a benign lesion, but often causes disturbances in the eruption of its associated tooth.^5,6^

This paper discusses a case of an unerupted left maxillary permanent central incisor due to the presence of complex odontome in a young patient with clinical presentation, radiographic features, histopathological features and surgical removal of odontome.

## CASE REPORT

A 12-year-old female patient reported with a complaint of missing tooth in the upper front region of the jaw. Past family and medical histories were not relevant. Her general medical history was noncontributory.

**Fig. 1:**
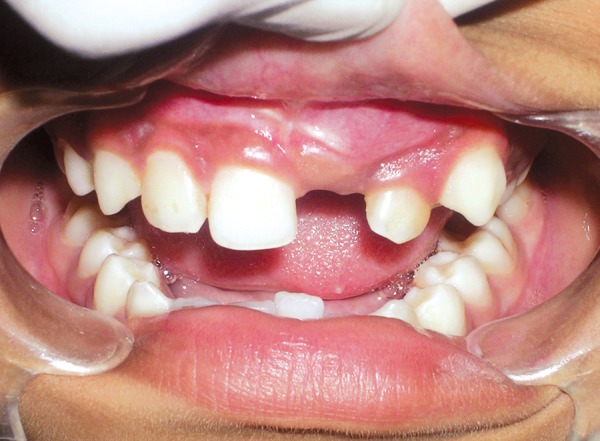
Intraoral view of the patient showing unerupted left maxillary permanent central incisor with a slight bulge

Intraoral examination revealed a permanent dentition with unerupted left maxillary permanent central incisor. On inspection a swelling was noticed on the labial side of the unerupted tooth (Fig. 1). A firm nodule measuring approximately 2 cm in diameter in the same region was palpated.

**Fig. 2:**
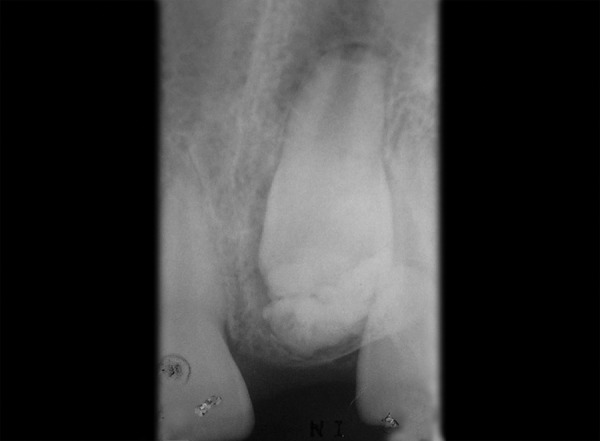
Intraoral periapical radiograph showing multilocular radiopacity enveloping the unerupted incisor with incomplete root formation

**Fig. 3:**
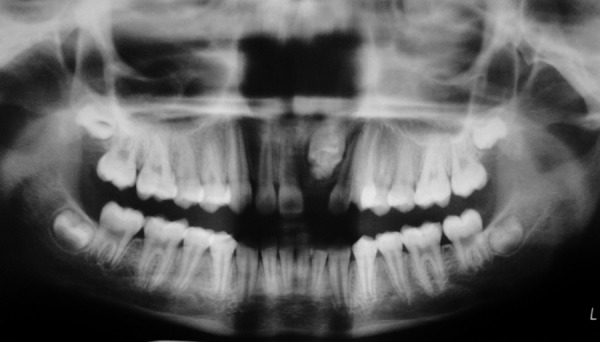
Preoperative panoramic radiograph showing multilocular radiopacity

Intraoral periapical and panoramic radiographs revealed that radiopaque structures were present obstructing the eruption of left maxillary permanent central incisor (Figs 2 and Figs 3). On basis of clinical and radiographic findings, case was provisionally diagnosed as odontome. Treatment consisted of surgical removal of the odontome and bonding of begg's bracket (along with a twisted ligature wire in a hook form) to the unerupted tooth for application of orthodontic traction if required.

**Fig. 4:**
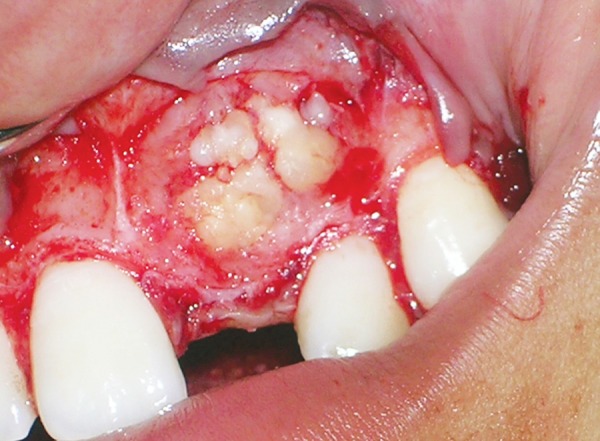
Surgical exposure of the lesion showing numerou calcified masses

**Fig. 5:**
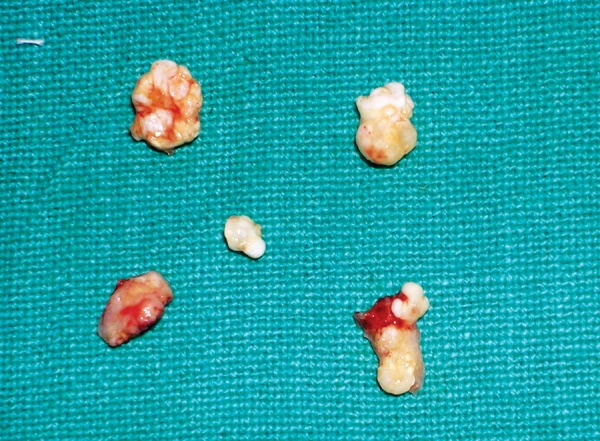
Excised calcified masses

**Fig. 6:**
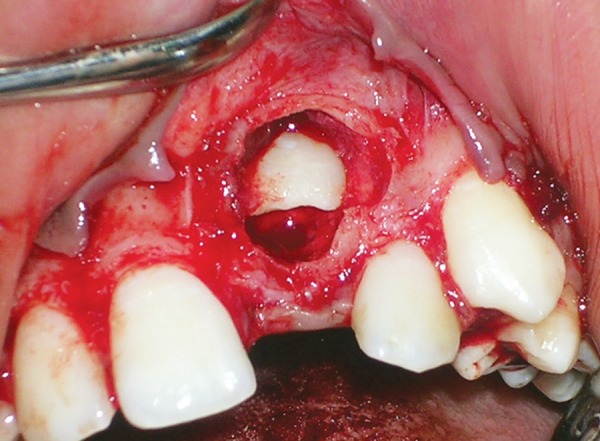
Exposure of the unerupted central incisor after complete removal of calcified masses

**Fig. 7:**
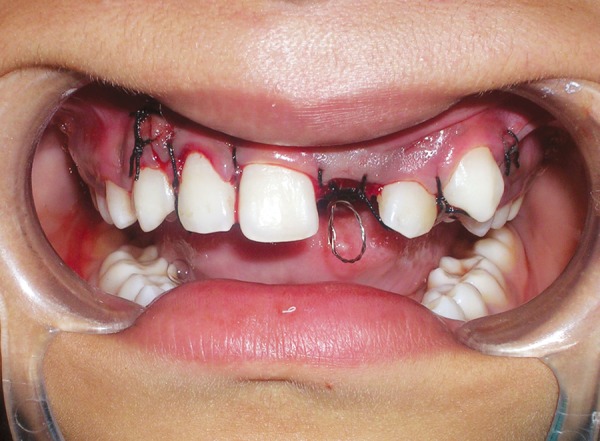
Flap repositioned and sutured with ligature wire hook suspended in the oral cavity

**Fig. 8:**
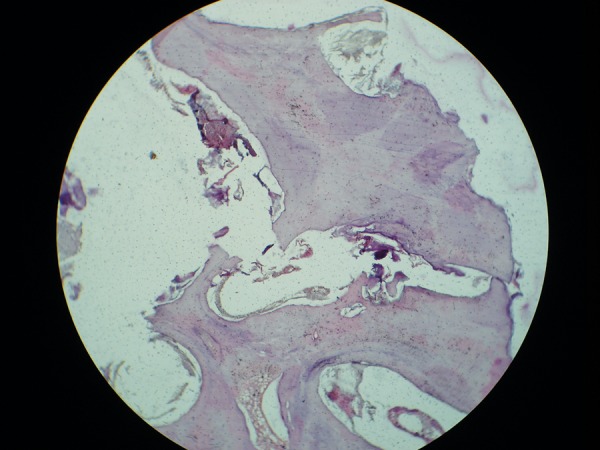
Microscopic section (H&E stained) exhibiting an irregular arrangement of dentin, mesenchymal tissue resembling pulp and a small area of cementum

The patient was subjected to surgical removal of the odontome under local anesthesia. Full thickness mucoperiosteal flap was reflected from the labial surface of right maxillary permanent lateral incisor to left maxillary permanent canine. The layer of bone overlying the mass was removed and the calcified masses were exposed (Fig. 4). The four to five calcified irregular masses were removed without disturbing the underlying tooth and send for histopathological examination (Fig. 5). Curettage was done and the area was irrigated with povidineiodine solution and normal saline (0.9%). Unerupted left maxillary permanent central incisor was located (Fig. 6) and after hemostasis, begg's bracket with a twisted ligature wire in a hook form tied to it was bonded on the labial surface of the impacted incisor. The flap was repositioned and sutured, keeping the ligature wire hook suspended in the oral cavity making sure the occlusion was not disturbed (Fig. 7). Microscopically, hematoxylin and eosin-stained (H&E) section showed structures exhibiting an irregular arrangement of dentin, mesenchymal tissue resembling pulp and a small area of cementum-like material (Fig. 8). Overall clinicopathological correlation was suggestive of complex odontome.

**Fig. 9:**
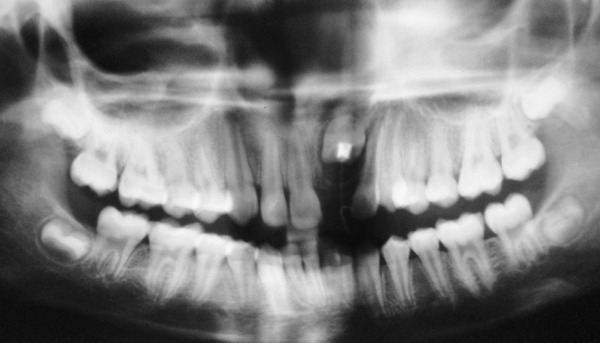
One week postoperative panoramic radiograph revealing proper placement of bracket and absence of any radiopaque masses

One week postoperative panoramic radiograph revealed the absence of radiopaque masses confirming the successful removal of the odontomes which obstructed the eruption of left maxillary permanent central incisor (Fig. 9).

## DISCUSSION

In clinical setting, dentists often encounter the problem of tooth impaction, which has been defined as a situation where a tooth fails to erupt into a normal functional position by the expected times.^7^ For tooth impaction, numerous local etiologic factors have been described. These include odontomes, odontogenic tumors, ankylosis, trauma and dentigerous cysts.^8-10^ Among these factors, odontoma is the most common etiological factor.

The term odontome by definition alone refers to a tumor of odontogenic origin. In a broad sense, it means a growth with both the epithelial and mesenchymal components exhibiting complete differentiation with the result that functional ameloblasts and odontoblasts form enamel and dentin. This enamel and dentin were usually laid down in an abnormal pattern because the organization of odontogenic cells failed to reach the normal state of morphodifferentiation.^11^ It was in 1867 that Paul Broca first used the term ‘odontome'.

According to WHO classification,^12^ odontomes can be divided into three groups:

1. *Complex odontome:* When the calcified dental tissues are simply arranged in an irregular mass bearing no morphologic similarity to rudimentary teeth.
2. *Compound odontome:* Composed of all odontogenic tissues in an orderly pattern that results in many teeth-like structures, but without morphologic resemblance to normal teeth.
3. *Ameloblastic fibro-odontome:* Consists of varying amounts of calcified dental tissue and dental papilla-like tissue, the later component resembling an ameloblastic fibroma. The ameloblastic fibro-odontome is considered as an immature precursor of complex odontome.

The exact etiology of odontome is unknown. However, it has been suggested that trauma and infection may lead to the development of such a lesion.^11^ It had been suggested by Hitchin, that odontomes are inherited or are due to a mutagene or interference, possibly postnatal, with the genetic control of tooth development.^13^

The etiology of odontome is that most result from extraneous odontogenic epithelial cells.^14^ When these buds are divided into several particles they may develop individually to become numerous, closely positioned malformed teeth or tooth-like structures. When the buds develop without such uncommon division and consists of haphazard conglomerates of dental tissues, they may develop into complex odontomes. However, the transition from one type to another is commonly associated with varying degrees of morphodifferentiation or histo-differentiation or both, and it is often difficult to differentiate between both the types.^14^

The treatment for odontomes in both primary and permanent dentition is their surgical removal. If odontomes are extirpated early without disturbing the underlying tooth germ, the eruption of the impacted teeth can then be expected spontaneously or after orthodontic traction.^2,7,8^ However, underlying impacted teeth are sometimes extracted in association with the removal of odontomas.^15^

In this case, the overlying odontomes were surgically removed and the impacted central incisor has been kept under observation to monitor its eruption. If the root of the impacted tooth is still developing, the tooth may erupt normally; but, once the root apex has closed, the tooth has lost its potential to erupt.^16^ Interestingly, orthodontic therapy is not usually necessary and is not applied to improve the malocclusion caused by odontome after extirpation of the tumor.^17^ The reason is that most odontomes are very small, and the influence of the tumor on occlusion might be improved without orthodontic therapy. Hisatomi et al^18^ reported that, the impacted tooth tended to erupt regardless of the degree of root formation after extirpation of the odontoma interfering with tooth eruption, although some teeth showed infraversion and/or crowding. In the case presented, root formation of the impacted incisor was not complete. Therefore, it is anticipated that impacted left maxillary permanent central incisor may erupt spontaneously. However, there have also been some reports about orthodontic therapy which might lead the impacted permanent tooth to a satisfactory postoperative occlusion. In the present case, if the eruption does not occur within 2 to 3 months, orthodontic traction will be done making use of the ligature wire hook on the bonded bracket. If the tooth does not erupt, suspended ligature wire hook will be used for orthodontic traction with no necessity for repeated flap reflection.

## CONCLUSION

Radiographic examination of all pediatric patients that present clinical evidence of delayed permanent tooth eruption or temporary tooth displacement, with or without history of previous dental trauma should be performed. Early diagnosis of odontomes allows adoption of a less complex and less expensive treatment and ensures normal eruption pattern of permanent teeth which may otherwise get impacted.
